# A Rare Case of Progressive Familial Intrahepatic Cholestasis Type 4: A Case Report and Literature Review

**DOI:** 10.7759/cureus.47276

**Published:** 2023-10-18

**Authors:** Hana Halabi, Khawla Kalantan, Warif Abdulhaq, Habeib Alshaibi, Mohammed A Almatrafi

**Affiliations:** 1 Department of Pediatrics, Maternity and Children Hospital, Makkah, SAU; 2 Department of Medicine and Surgery, Medical College of Umm Al-Qura University, Makkah, SAU; 3 Department of Anatomic Pathology, Maternity and Children Hospital, Makkah, SAU; 4 Department of Pediatrics, Umm Al-Qura University, Makkah, SAU

**Keywords:** case report, pfic4, progressive familial intrahepatic cholestasis, intrahepatic cholestasis, hyperbilirubinemia during infancy

## Abstract

Progressive familial intrahepatic cholestasis (PFIC) is a group of genetic disorders characterized by progressive intrahepatic cholestasis. Different mutations in hepatocellular transport genes result in distinct PFIC subtypes with unique clinical manifestations, laboratory findings, and histopathological characteristics. Three PFIC genotypes have been commonly described (PFIC 1, 2, and 3), but in recent years, PFIC 4, 5, and 6 genetic mutations have been identified. Here, we report the first PFIC 4 case in the Middle East in a 46-day-old male infant who was successfully treated with a liver transplant. A 46-day-old, male, full-term infant presented with persistent jaundice and obstructive liver pathology suggested by liver profile and biopsy. Whole exome sequencing confirmed the diagnosis of PFIC 4. Medical treatment failed to improve the patient’s symptoms. Therefore, the patient underwent hepatectomy and an unrelated liver transplant. He is currently exhibiting significant clinical improvements and is free of active complaints. PFIC is a rare disease that poses diagnostic and therapeutic challenges for clinicians. Infants presenting with unexplained cholestasis should have PFIC 4 as a differential diagnosis. Early recognition and treatment of PFIC 4 with liver transplantation may result in a more favorable prognosis.

## Introduction

Cholestasis is a clinical condition that results from the impairment of bile flow. Numerous mutations and pathways associated with cholestasis have been described [1].

Progressive familial intrahepatic cholestasis (PFIC) encompasses a heterogeneous group of liver disorders of autosomal recessive inheritance caused by bile secretion defects [2]. The exact incidence of PFIC is unknown; however, it accounts for 10% to 15% of pediatric cholestasis, with an equal distribution between males and females [3]. These defects are caused by mutations affecting genes that encode proteins expressed in the apical membrane of the hepatocyte that are involved in hepatocellular transport [1,2].

Historically, they were classified into three subtypes based on their clinical presentation, laboratory results, liver histopathological findings, and genetic defects [2]. Genetic mutations in the ATP8B1 gene encoding the FIC1 protein, the ABCB 11 gene encoding the BSEP protein, and the ABCB4 gene encoding the MDR3 protein cause PFIC types 1, 2, and 3, respectively [2].

In recent years, numerous novel variants of PFIC have been identified [4]. Sambrotta et al. described the first case of PFIC type 4 (PFIC4) in 2014 [5]. PFIC4 is associated with the gene that encodes TJP2 (tight junction protein-2). TJP2, referred to as zona occludens 2 (ZO-2), stabilizes the junction between the cytoskeleton actin and the cytoplasmic C termini proteins like claudins [6]. There is a tight junction formed by the hepatocyte basal membrane surface and the bile duct canaliculi at the junction of the bile duct canaliculi and the hepatocyte basal membrane surface that separates bile from plasma [5]. Mutations in the TJP2 gene can cause defects in tight junction function, resulting in severe cholestatic liver disease with extrahepatic symptoms [5].

There are few case reports of PFIC 4 to date [5,7-11]. We report the first PFIC 4 case in the Middle East involving a 46-day-old male infant with chronic cholestatic liver disease and low gamma-glutamyltransferase (GGT) diagnosed with TJP2-related PFIC 4 and treated with a liver transplant.

## Case presentation

A 46-day-old, male, full-term infant came to our hospital with persistent jaundice, clay-colored stool, and dark urine. He was admitted to the neonatal intensive care unit (NICU) for four days due to neonatal jaundice that required phototherapy. Following his discharge from the NICU, he was lost to follow-up. His parents, however, reestablished his medical care at our institution after one month due to parental concerns about persistent jaundice. He has been thriving well with good oral intake and activity. Apart from consanguineous marriage, his family history was unremarkable.

He appeared well, with normal vital signs for his age. His growth parameters were normal for his age (weight: 5.3 kg at the 63.6 percentile; length: 58 cm at the 69.7 percentile). His sclera and skin were both deeply jaundiced. His physical examination was negative for dysmorphic features. The abdomen was soft and lax, with no distention or organomegaly. Systemic examinations show an open anterior fontanelle (AF), normal tone, power, and reflexes, normal S1 and S2, no added sound, equal bilateral air entry (EBAE), no bruises, or skin rashes, normal male genitalia, circumcised, bilateral descended testes.

On initial workup (Table 1), the liver profile was suggestive of obstructive hepatic pathology, as evidenced by mild transaminitis (alanine aminotransferase (ALT): 288 IU/L; aspartate aminotransferase (AST): 270 IU/L), direct hyperbilirubinemia (total bilirubin: 164.7 umol/L, direct bilirubin: 118.3 umol/L), and elevated alkaline phosphatase (ALP: 927 IU/L), normal GGT: 34 IU/L, and high alpha-feto protein (1832.3 ng/ml). Synthetic liver function tests for albumin and coagulation profile were within normal limits (albumin: 37 g/L; partial thromboplastin time (PTT): 37.5 seconds; prothrombin time (PT): 11.0 seconds; and international normalized ratio (INR) 1).

**Table 1:** Serial liver function AST: aspartate aminotransferase; ALT: alanine aminotransferase; ALP: alkaline phosphatase; GGT: gamma-glutamyl transferase

		At the time of presentation	Pre liver transplantation	Post liver transplantation	Reference range	
Bilirubin	Total	164 umol/L	301 umol/L	20.6 umol/L	≤88.4 umol/L	
	Direct	118 umol/L	208 umol/L	8 umol/L	0-26.5 umol/L	
AST		288 IU/L	381 IU/L	32 IU/L	8-60 IU/L	
ALT		270 IU/L	291 IU/L	38 IU/L	7-55 IU	
ALP		927 IU/L	1102 IU/L	399 IU/L	122-469 IU/L	
GGT		34 U/L	28 U/L	21 U/L	0-11 months	1-6 years
					<178 U/L	<21 U/L
Alpha-fetoprotein		1832 ng/ml	2002.82 ng/ml	-	<8.4 ng/ml	

Ultrasound examination of the abdomen revealed a normal biliary system and a normal liver in terms of size, shape, and homogeneity.

**Figure 1:**
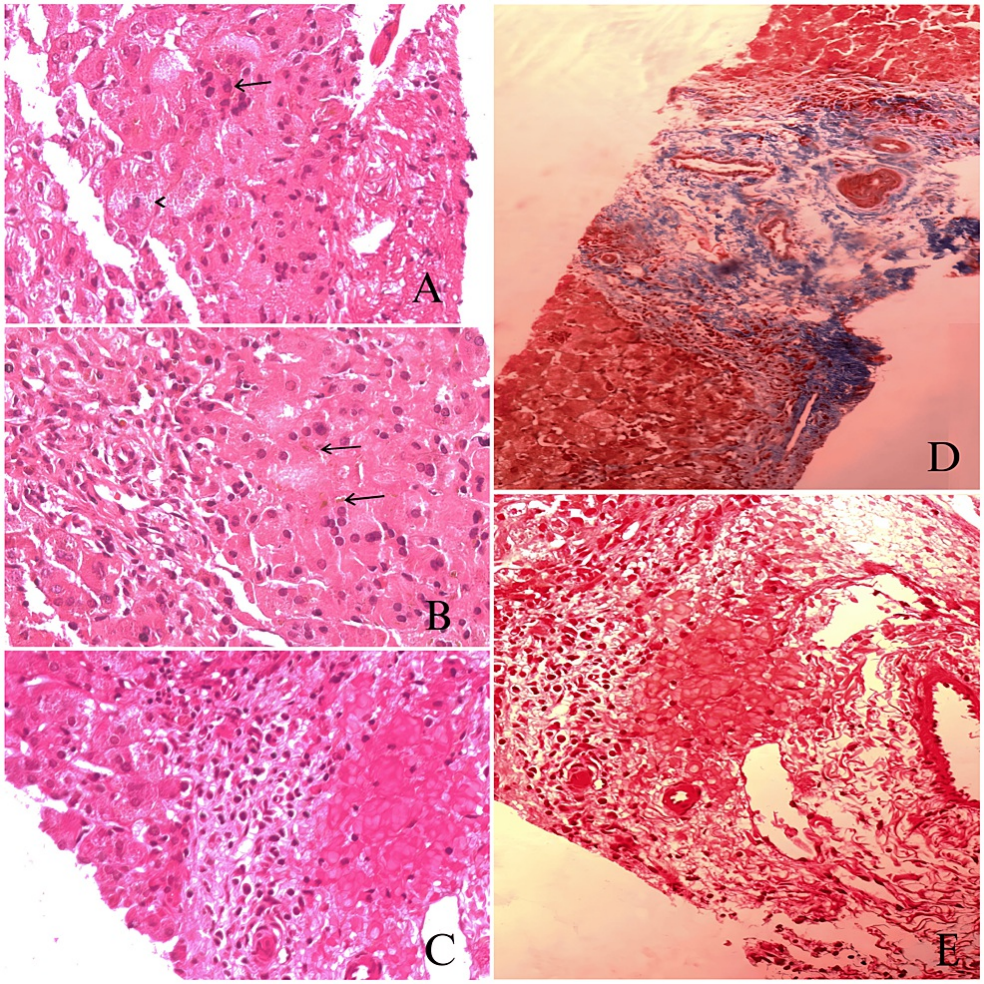
Liver histopathology (at the age of two months) A. Arrow: giant cell, arrowhead: ballooning. B. Arrow: cholestasis. C. Portal inflammation. D: fibrosis stage 2. E: higher picture of portal inflammation.

Histopathological examination of the liver biopsy specimen revealed focal loss of main bile ducts, marked cholestasis with portal and peri-portal inflammation (grade 2), as well as fibrosis (stage 1), and paucity of intrahepatic bile ducts (Figure 1).

Cholangiography performed intraoperatively revealed a patent bile duct, no inclusion defect in the gall bladder, and no biliary system dilatation.

Whole exome sequencing revealed homozygous mutations in exon 17 of TJP2 (+) (C.2524C >T), confirming the diagnosis of PFIC 4. Additionally, two regenerative liver nodules are identified with no evidence of dysplasia or malignancy.

At the age of one month, the patient was started on ursodeoxycholic acid (UDCA) for symptomatic relief; however, on subsequent follow-up visits over a five-month period, the patient continued to have persistent cholestasis, severe pruritus, and worsening liver function test parameters (total bilirubin: 600, direct bilirubin: 500, INR: 2).

The patient underwent hepatectomy and an unrelated liver transplant without complications at 11 months. He is currently exhibiting significant clinical improvements and is free of active complaints.

## Discussion

PFIC is a group of rare inherited liver diseases that progress to cirrhosis and end-stage liver disease [3]. Genetically confirmed PFIC has several variants; types 1 and 2 are among the most widely prevalent varieties, representing two-thirds of PFIC cases, followed by type 3, which represents one-third of patients [3]. Subsequently, a newer variant of PFIC has been identified, known as PFIC 4 [4].

In the diagnosis of PFIC diagnosis, the onset of the disease, extrahepatic manifestations, GGT level, and molecular and genetic testing are all crucial for determining the variant type [12]. Cholestasis onset in the neonatal period or early infancy are characteristics of PFIC 1 and 2, whereas patients with PFIC 3 may manifest late in infancy, childhood, or adolescence [13]. Extrahepatic manifestations of PFIC1 include persistent short stature, sensorineural hearing loss, persistent watery diarrhea, cholecystitis, pancreatitis, and elevated sweat electrolyte concentration, whereas PFIC2 has not been associated with any extrahepatic symptoms [1]. Early onset of PFIC3 is associated with stunted growth, osteopenia, and learning disabilities [13]. However, late-onset PFIC 3 in late childhood and adolescence commonly presents with gastrointestinal bleeding secondary to portal hypertension and liver cirrhosis [13]. Regarding GGT levels, PFIC 1 and 2 are characterized by low GGT levels while patients with PFIC 3 have an elevated GGT level [1].

Based on clinical presentation, liver biopsies, and molecular testing, our patient was diagnosed with PFIC 4. Few reported cases were available in the literature [5,7-11]. The clinical manifestations of PFIC 4 in the neonatal period range from mild neonatal cholestasis and obstructive jaundice [4] to a more severe clinical course [4,5,7]. Nida Mirza et al. reported a case of PFIC 4 with on-and-off mild pruritus [14]. However, other different cases present with severe progressive liver disease, intense pruritus, and liver failure [4,5,7]. Similar to these cases, our patient presented with unexplained cholestasis, severe pruritis, and worsening liver function. Our patient's GGT level remained normal throughout the disease course. This observation has been reported in the literature in patients diagnosed with PFIC4 [4]. Few patients with PFIC4 exhibit neurological and respiratory symptoms, but our patient did not demonstrate extrahepatic manifestations in his clinical presentation [13].

The clinical course and progression to liver cirrhosis vary between PFIC subtypes [1]. In contrast to PFIC type 3, PFIC types 1 and 2 have rapid progression and are strongly associated with hepatocellular carcinoma (HCC) [15,16]. Despite the scarcity of reported cases of PFIC 4 in the available literature, Zhou et al. reported two patients with PFIC 4 who developed HCC [7]. Both patients were presented with neonatal jaundice, normal GGT, and elevated alpha-fetoprotein levels. This report indicates that PFIC4 may be associated with an increased risk of HCC, necessitating close monitoring and early liver transplantation for a better prognosis. The association between PFIC4 and HCC may be caused by somatic mutations and gene amplifications in children with bile salt export pump deficiency such as TJP2 deficiency [17].

PFIC 4 treatment has both medical and surgical treatment options. Zhang et al. reported that seven patients diagnosed with PFIC 4 responded favorably to medical treatment. Similar to Zhang et al., Nida Mirza et al., and Ting Ge et al. both reported a case of PFIC 4 that had a stable chronic liver disease and controlled symptoms with medical therapy [8,14]. However, Sambrotta et al. reported that nine out of 12 (75%) published case series underwent liver transplantation after failure of medical therapy. Most patients reported in the study had favorable prognoses following liver transplantation. Two patients had mild portal hypertension, one with recurrent unexplained hematoma, and only one reported mortality [5]. Our patient received a liver transplant following the failure of medical treatment, worsening pruritus, and an abnormal liver function test. Our patient's clinical symptoms and liver function abnormalities have resolved, and he has been doing well since receiving a liver transplant.

## Conclusions

PFIC is a rare disease that poses diagnostic and therapeutic challenges for clinicians. Infants presenting with unexplained cholestasis should have PFIC 4 as a differential diagnosis. Early recognition and treatment of PFIC 4 with liver transplantation may result in a more favorable prognosis.
